# A Prescriptive Model for Protecting Nurses' Dignity and Safety: Understanding Coping Processes in the Face of Workplace Violence

**DOI:** 10.1002/nop2.70557

**Published:** 2026-05-25

**Authors:** Zahra Ebrahimi Rigi, Parvin Mangolian Shahrbabaki, Fazlollah Ahmadi, Ali Ravari

**Affiliations:** ^1^ Department of Nursing, School of Nursing and Midwifery Iranshahr University of Medical Sciences Iranshahr Iran; ^2^ Nursing Research Center Kerman University of Medical Sciences Kerman Iran; ^3^ Department of Nursing, Faculty of Medical Sciences Tarbiat Modares University Tehran Iran; ^4^ Department of Medical Surgical Nursing, School of Nursing and Midwifery, Geriatric Care Research Center Rafsanjan University of Medical Sciences Rafsanjan Iran

**Keywords:** coping strategies, dignity, grounded theory, model, nurses, workplace violence

## Abstract

**Aims:**

This study aims to design a prescriptive model for protecting nurses' dignity and safety by systematically exploring their coping processes in response to workplace violence and examining how these empirically derived processes can inform the development of a conceptually grounded prescriptive framework.

**Design:**

A qualitative, two‐phase design was used. In Phase I, grounded theory based on Strauss and Corbin's approach explored nurses' coping processes and the mechanisms influencing dignity and safety in violent work settings. In Phase II, conceptual synthesis was performed using Walker and Avant's model development method to construct a prescriptive framework informed by and logically derived from the grounded findings.

**Methods:**

Data were collected through semi‐structured interviews with 39 nurses from hospitals in Kerman between June 2019 and December 2021. Given the extended data collection period, potential contextual influences, including the COVID‐19 pandemic, were considered during data interpretation. Theoretical saturation was achieved after 35 interviews, with subsequent interviews confirming the stability and completeness of emerging categories.

**Findings:**

The grounded theory study revealed that, in response to a main concern of perceived threats to dignity and job security within contextual conditions at multiple levels, nurses adopted action/interaction strategies (coping) involving adaptive, protective and meaning‐oriented approaches that shaped their perceptions of dignity and safety. This coping process was influenced by intervening conditions, including facilitators such as accountable and reassuring colleagues and supportive institutions and barriers such as managerial neglect and structural deficiencies. These coping strategies led to consequences characterised by divergent outcomes: adaptive responses fostered emotional recovery, confidence and motivation, whereas maladaptive responses resulted in burnout, anxiety and ongoing psychological distress. Building on these findings, the prescriptive model comprises three interrelated conceptual components: (1) Context Familiarisation (systematic assessment of risks and situational conditions), (2) Professional and Organisational Support (conceptual mechanisms of empowerment and resource alignment) and (3) Continuous Evaluation and Reinforcement (iterative monitoring of outcomes and refinement of responses). These components reflect an integrated process linking awareness, empowerment and evaluation, with each component grounded in participants' reported coping experiences. Rather than representing an empirically validated intervention, this framework conceptually outlines organisational responsibilities across different levels, thereby strengthening the conceptual coherence of the model. This framework translates empirical insights into a structured yet adaptable form of guidance.

**Conclusions:**

The proposed model offers a conceptually grounded framework that outlines key organisational mechanisms through which healthcare organisations can strengthen nurses' capacity to preserve dignity and safety in the face of workplace violence. However, the model remains interpretive and context‐sensitive and has not undergone empirical validation. While the model provides theoretically informed and practice‐oriented directions, further empirical testing is required. By providing a structured framework for the design, implementation and evaluation of comprehensive violence prevention systems, the model supports, rather than confirms, organisational action.

**Implications for the Profession and/or Patient Care:**

By operationalising dignity and safety as explicit organisational outcomes, the model provides a practical framework for developing culturally sensitive, context‐specific interventions. However, some strategies may require adaptation across different healthcare contexts to ensure broader applicability. Implementing these prescriptive strategies may contribute to enhancing nurses' well‐being, retention and quality of patient care.

**Reporting Method:**

The study followed COREQ guidelines.

**Impacts:**

Despite ongoing prevention initiatives, workplace violence against nurses persists, undermining their dignity and safety. The proposed prescriptive model offers a structured pathway for translating nurses' coping experiences into conceptually grounded organisational and policy actions. For nursing managers, it emphasises the institutionalisation of supportive mechanisms that may enhance resilience and strengthen reporting practices. For policymakers, it highlights the need to embed dignity and safety indicators within national standards for workplace violence prevention. Interventions must remain context‐sensitive—responsive to cultural norms, clinical environments and crisis conditions such as the COVID‐19 pandemic—and undergo continuous evaluation and refinement. Future research should empirically test and validate the model across diverse healthcare settings to strengthen its generalisability and practical utility.

**Patient or Public Contribution:**

No patient or public contribution.

## 1 Introduction

Workplace violence (WPV) has emerged as one of the most pervasive occupational hazards in healthcare, encompassing physical assaults, verbal abuse, psychological intimidation and sexual harassment (Byon et al. 2022). The World Health Organization (WHO) and the International Labour Organization (ILO) recognise WPV as a critical psychosocial risk factor that undermines both individual and organisational well‐being. Among healthcare professionals, nurses are particularly vulnerable because of their sustained and close contact with patients, unpredictable work shifts and emotionally charged care environments (Liu et al. 2019; Hacer and Ali 2020). Consequently, the National Institute for Occupational Safety and Health (NIOSH) identifies nursing as one of the most stressful professions, with repeated exposure to violence contributing to burnout, absenteeism and turnover (Sinclair et al. 2023). While numerous studies have examined the prevalence, predictors and consequences of WPV, most have focused narrowly on incident reporting or individual coping behaviours (d'Ettorre et al. 2018), with comparatively limited attention to the underlying processes through which nurses interpret, negotiate and respond to such experiences in practice.

Although workplace violence against nurses is a well‐documented phenomenon, evidence indicates that it remains a persistent problem worldwide (García‐Pérez et al. 2021). However, the relational and ethical dimensions of violence—specifically its implications for nurses' dignity and safety—remain underexplored. Dignity refers to the intrinsic worth and professional respect that enable nurses to act autonomously and ethically within their roles, whereas safety denotes both physical protection and psychological security necessary for effective practice (Zhang et al. 2021). When these are compromised, the consequences extend beyond personal well‐being to affect patient safety, teamwork and the moral climate of healthcare institutions.

Existing models of coping or violence prevention tend to be descriptive or reactive, focusing on documenting experiences rather than providing structured, context‐sensitive guidance for action. Moreover, the transition from empirical insights into coherent, conceptually grounded prescriptive frameworks remains insufficiently articulated in the literature. This gap highlights the need for a prescriptive model that not only synthesises empirical findings but also translates them into actionable and theoretically coherent guidance. There remains a critical need for a prescriptive model that integrates nurses' lived coping processes with organisational mechanisms designed to sustain dignity and safety under conditions of workplace violence.

In response to this conceptual and practical gap, the present study proposes a contextually grounded prescriptive model that is explicitly derived from grounded theory findings and conceptually structured to link awareness, empowerment and evaluation processes. The model is intended as an interpretive and practice‐oriented framework rather than an empirically validated solution, delineating how nurses may preserve dignity and safety while managing workplace violence and offering preliminary guidance for nursing management and policy development.

## 2 Background

Workplace violence (WPV) encompasses a spectrum of harmful behaviours—including verbal abuse, bullying, physical assault and intimidation—that occur in work‐related contexts (Salin et al. 2018). Its definitions and manifestations are culturally mediated, complicating efforts to develop universal prevention strategies (Leather et al. 2021). Nurses face heightened risk as staffing shortages, organisational culture and the nature of direct patient care create environments where aggression thrives (Byon et al. 2022). Critically, WPV is not only a threat to physical safety but also a profound assault on professional dignity—undermining respect, contributing to moral distress and challenging nurses' sense of professional identity. These violations directly undermine psychological well‐being, job satisfaction and ultimately, the capacity to provide ethical, high‐quality care (Shi et al. 2020; Zhang et al. 2021).

Despite recognition of WPV as a global occupational hazard and the implementation of common countermeasures (e.g., reporting systems, staff training), prevalence remains unacceptably high (García‐Pérez et al. 2021). These interventions often fail due to systemic underreporting, lack of managerial support and a generalised, non‐contextualised approach that neglects specific cultural and organisational realities (Spelten et al. 2020). Frequently, the burden of response is placed on individual nurses through resilience or de‐escalation training, rather than on rectifying the organisational dynamics that perpetuate violence. Consequently, nurses must rely heavily on personal, often unsupported, coping mechanisms (Pariona‐Cabrera et al. 2020), highlighting a misalignment between individual‐level expectations and organisational responsibilities.

Coping strategies—the cognitive and behavioural patterns used to manage stressors—are thus central to how nurses navigate violent workplaces (Abouammoh et al. 2020). When coping fails or is overwhelmed, severe negative outcomes ensue, including burnout, emotional distress and withdrawal from the profession. However, existing literature tends to categorise coping responses without adequately explaining the dynamic and context‐dependent processes through which such strategies are developed, adapted and sustained within specific organisational environments.

While the prevalence and consequences of WPV are well‐documented, fewer studies have qualitatively examined the processes by which nurses interpret, navigate and cope with these events in their specific contexts. This gap is particularly acute in non‐Western settings, where social hierarchies and cultural norms profoundly influence both the experience of violence and the repertoire of coping responses (Combrinck et al. 2022). Existing frameworks are largely descriptive, cataloguing types of violence or correlating them with outcomes. What is missing is a prescriptive model—one that synthesises an understanding of nurses' lived coping experiences into a structured, conceptually grounded framework to inform organisational responses. Importantly, such a model should remain conceptually oriented rather than overly procedural to ensure transferability across settings. Accordingly, the aim is not to provide a universally applicable solution but to offer an interpretive framework that can be adapted to different contexts. Such a model is needed to translate insight into intervention, moving beyond generic policies to provide healthcare organisations with a conceptually informed and context‐sensitive basis for action.

This study addresses this need. Using grounded theory methodology to explore the coping processes of Iranian nurses, thereby replacing previously anonymised contextual information, this research aims to develop a prescriptive model that is conceptually derived from participants' experiences and analytically constructed to outline key organisational and professional processes to support nurses' dignity and safety. The model is not intended as an empirically validated intervention but as a theoretically informed framework requiring further testing across diverse healthcare settings.

## 3 The Study

### 3.1 Aims

This study was designed to explore nurses' coping processes in response to workplace violence and to develop a conceptually grounded prescriptive model informed by these processes.

Two research questions were addressed.

1. What is the process through which nurses cope with workplace violence?
2. How can the empirically derived coping processes be translated into a prescriptive model that conceptually informs organisations in supporting nurses' dignity and safety?

## 4 Methods

### 4.1 Design and Setting

A sequential, two‐phase qualitative design was conducted across multiple hospitals in Kerman, Iran, between June 2019 and December 2021. Given that part of the data collection overlapped with the COVID‐19 pandemic (2020–2021), potential influences on workplace conditions, patient load and the incidence or perception of workplace violence were explicitly considered during data interpretation.

- *Phase One (June 2019–May 2021)* employed grounded theory methodology based on Corbin and Strauss's approach to inductively explore nurses' coping processes in response to workplace violence and associated experiences of dignity and safety (Corbin and Strauss 2015).
- *Phase Two (mid‐2021–December 2021)* applied Walker and Avant's theory synthesis approach to translate the grounded theory into a conceptually grounded prescriptive model, emphasising interpretive guidance for organisational decision‐making and interventions rather than presenting prescriptive procedures. (Walker and Avant 2019).

### 4.2 Logic of Theory Development

This study did not apply a pre‐established theoretical framework to guide data collection. Instead, a theory‐generating approach oriented toward model development was adopted, consistent with Meleis's theory–research–theory cycle (Meleis 2018). Theory development progressed iteratively from:

1. *Description*: Capturing nurses' lived experiences,
2. *Explanation*: Analysing how and why coping unfolds and
3. *Prescription*: Identifying conceptually grounded organisational mechanisms to support nurses' dignity and safety.

This explicit progression distinguishes the resulting model from purely descriptive or explanatory frameworks. However, in line with grounded theory principles, the prescriptive component is conceptually derived from empirical findings rather than framed as a set of procedural or mandatory policy directives. The final model is prescriptive because it articulates principle‐based, conceptually grounded guidance, rather than fixed procedures, including suggested organisational actions at multiple levels (individual, managerial and institutional) intended to safeguard nurses' dignity and safety.

### 4.3 Sample and Recruitment

Consistent with grounded theory methodology, purposeful sampling followed by theoretical sampling was used based on Corbin and Strauss's approach (Corbin and Strauss 2015). Initial purposeful sampling included 31 clinical nurses selected to ensure maximum variation in age, gender, clinical unit and work experience. As analysis progressed, theoretical sampling guided the recruitment of eight additional key informants (e.g., head nurses, supervisors and a physician) to elaborate emerging categories, particularly those related to organisational support and power dynamics.

Sampling continued until theoretical saturation was achieved, defined as the point at which no new conceptual properties emerged and relationships among categories were fully developed and validated within the data. The final sample consisted of 39 participants (32 women and 7 men), aged 23–47 years, with 6 months to 24 years of professional experience. Written informed consent was obtained from all participants prior to data collection.

### 4.4 Data Collection

Data were collected through 39 semi‐structured, face‐to‐face interviews, supplemented with field notes. Interviews lasted 30–90 min and were conducted in private hospital settings. Initial interview questions were broad (e.g., ‘Can you describe your experience of workplace violence?’), followed by focused prompts addressing coping processes (e.g., ‘How did you respond?’, ‘What helped or hindered you?’). Probing questions were used to deepen reflection and clarification. All interviews were audio‐recorded with consent and transcribed verbatim. Field notes documented contextual observations, emotional responses and reflexive insights, enhancing analytic depth. Data collection continued until theoretical saturation was achieved after 35 interviews; the remaining four interviews confirmed the stability and completeness of categories.

### 4.5 Data Analysis

#### 4.5.1 Construction of a Descriptive Theory (Phase One)

Data collection and analysis proceeded concurrently from June 2019 to May 2021, guided by the constant comparative method within Strauss and Corbin's grounded theory approach, which consists of five steps: open coding for concept identification, developing concepts (properties and dimensions), contextual analysis of conditions, interactions and consequences, integrating process into the analysis and integrating categories to form the theory (Corbin and Strauss 2015). The analysis progressed through three coding phases:

*Open Coding (Step 1)*: Transcripts were reviewed repeatedly to extract meaningful units and identify initial concepts related to nurses' experiences of workplace violence.

*Axial Coding (Steps 2–4)*: Codes were continuously compared, refined and grouped based on similarities and differences. This phase involved exploring conceptual dimensions, determining contextual factors across individual (microsystem), organisational (mesosystem) and societal (macrosystem) levels and identifying strategies employed by participants in response to the phenomenon. The evolving categories were examined for their relationships to form a coherent analytical structure.

*Selective Coding (Step 5)*: Relationships among categories were systematically integrated to develop a coherent theoretical framework explaining nurses' coping processes.

#### 4.5.2 Development of the Prescriptive Model (Phase Two)

The prescriptive model was developed using Walker and Avant's theory synthesis approach, which involves the systematic integration of empirically derived concepts with existing theoretical and empirical knowledge to produce a conceptually grounded framework. This approach was selected because it is specifically intended for constructing prescriptive theories that provide principle‐based guidance for professional action. The synthesis process included three key steps:

1. *Adopting the core grounded theory concept*: ‘protecting nurses' dignity and safety in the face of workplace violence’.
2. *Conducting an integrative literature review* (2001–2022) to identify relevant concepts, interventions and relational patterns.
3. *Creative synthesis*: Systematically linking empirical coping processes with higher‐level organisational, educational and policy mechanisms (e.g., awareness, empowerment, evaluation) rather than context‐specific operational actions. The model specifies suggested actions, by whom and at which organisational level, while maintaining conceptual guidance and theoretical coherence.

### 4.6 Ethical Considerations

This study received ethical approval from the Kerman University of Medical Sciences Ethics Committee (Code: IR.KMU.REC.1398.174). All procedures complied with the Declaration of Helsinki. Participants were informed of their rights, assured of confidentiality and informed of their right to withdraw at any time. Identifying information was replaced with codes to ensure anonymity.

### 4.7 Rigour and Validation of the Prescriptive Model

Trustworthiness was ensured using Guba and Lincoln's criteria (credibility, dependability, confirmability and transferability) (Vu 2021). Several strategies were employed to enhance credibility, including prolonged engagement with the data and continuous monitoring by the research team. Field notes and memos were used to gather supporting evidence. Member checking was performed, with participants reviewing some analysed data to ensure that results accurately reflected their experiences.

To validate the prescriptive model, a formal expert review was conducted. Three senior nursing scholars with expertise in qualitative research, theory development and occupational health reviewed the preliminary model. Feedback focused on conceptual coherence, feasibility and applicability. Peer debriefing sessions within the research team further refined relationships among model components, ensuring that the final model was theoretically robust while remaining conceptually interpretable across diverse clinical settings.

However, the limited number of experts involved in the validation process represents a methodological constraint and broader validation approaches (e.g., Delphi panels or stakeholder engagement) are recommended for future research to strengthen the model's robustness and generalisability.

## 5 Findings

### 5.1 Grounded Theory (First Phase)

Using constant comparative analysis, approximately 1500 initial codes were condensed into 17 subcategories, which were further integrated into five supporting categories organised around a single core category (see Table 1). This process resulted in a grounded theory that conceptually explains how nurses cope with workplace violence while striving to preserve their dignity and job security.

**TABLE 1:** Analytic Structure of the Grounded Theory.

Core category	Supporting categories	Subcategories
Protecting nurses' dignity and safety in violent work environments	Contextual conditions	*Individual level*: Limited experience, interpersonal vulnerability
		*Organisational level*: Workload pressure, insufficient support
		*Societal level*: Normalisation of violence, weak protections
	Main concern	Perceived loss of respect and professional value
		Emotional exhaustion and insecurity
	Action/interaction strategies (coping)	*Adaptive coping*: Emotional regulation, teamwork and support, situational management, meaning‐based coping
		*Maladaptive coping*: Avoidance, silence, retaliatory responses
	Intervening conditions	*Facilitators*: Supportive colleagues, responsive institutions
		*Barriers*: Managerial inaction, structural constraints
	Consequences	*Constructive outcomes*: Recovery, resilience, professional growth
		*Adverse outcomes*: Burnout, distress, continued exposure to violence

#### 5.1.1 Core Category: Protecting Nurses' Dignity and Safety in Violent Work Environments

The core category captures the central social process underlying participants' experiences. Workplace violence was consistently interpreted as a threat to both professional dignity (e.g., loss of respect, moral distress, identity erosion) and job security. Protecting these elements emerged as the primary concern guiding nurses' coping strategies.

#### 5.1.2 Supporting Categories

##### 5.1.2.1 Contextual Conditions at Multiple Levels

*Individual‐level:* Factors such as limited experience and fragile interpersonal relationships.

It was a busy shift… the shift manager shouted at me in front of the supervisor and said, ‘You don't understand that you should have informed me about shortages’. (Participant 19).

The trainee nurses… complied with all unreasonable demands…allowing the experienced staff to continue being bossy and humiliating us. (Participant 10).

*Organisational‐level:* Heavy workloads, staff shortages and insufficient institutional support.

Our number was low; the ward was busy… one of the patients started swearing and shouting. (Participant 27).

In the burn ward… experienced staff became irritable…someone started yelling and being aggressive toward me. (Participant 33).

*Societal‐level:* Cultural permissiveness toward violence and weak legal protections.

They think if we laugh together, we are doing something wrong… it really hurt me. (Participant 34).

A surgical resident…mocked my attempt to ensure patient consent. (Participant 27).

##### 5.1.2.2 Main Concern: Perceived Threat to Dignity and Job Security

Participants emphasised that the main concern was the impact on dignity, safety and professional identity rather than the violent act itself.

After these incidents, I kept asking myself why I chose nursing. (Participant 32).

I've been slapped, pushed and sexually harassed… Now I just do basic care… it doesn't have to be thorough. (Participant 30).

##### 5.1.2.3 Action/Interaction Strategies (Coping)

*Adaptive coping:* Emotional regulation, peer support, situational management and meaning‐making.

I accepted the situation and tried to grow stronger instead of breaking down. (Participant 22).

I go to the gym and swim…It helped me cope better. (Participant 1).

*Maladaptive coping:* Silence, avoidance, withdrawal, or retaliatory responses.

Frequent patient violence…led to unbearable pressure and self‐medication. (Participant 36).

Trainee nurses… tried to stay close to experienced staff and did everything they said… to avoid bullying. (Participant 14).

##### 5.1.2.4 Intervening Conditions

- Facilitators: supportive colleagues, responsive institutions.
- Barriers: managerial neglect, weak reporting systems, structural deficiencies.

We reported the behaviour many times, but no one followed up. (Participant 2).

Our payment list… was different from the Ministry's… they treated us very badly. (Participant 3).

##### 5.1.2.5 Consequences: Constructive Versus Adverse Outcomes

Coping strategies led to divergent outcomes:

*Maladaptive:* Burnout, anxiety, continued vulnerability.

*Adaptive:* Recovery, confidence and professional growth.

When I became stronger, my confidence returned. (Participant 5).

I work conscientiously, but they harass me because I don't give in to their bullying. (Participant 12).

#### 5.1.3 Descriptive–Explanatory Theory

**FIGURE 1:**
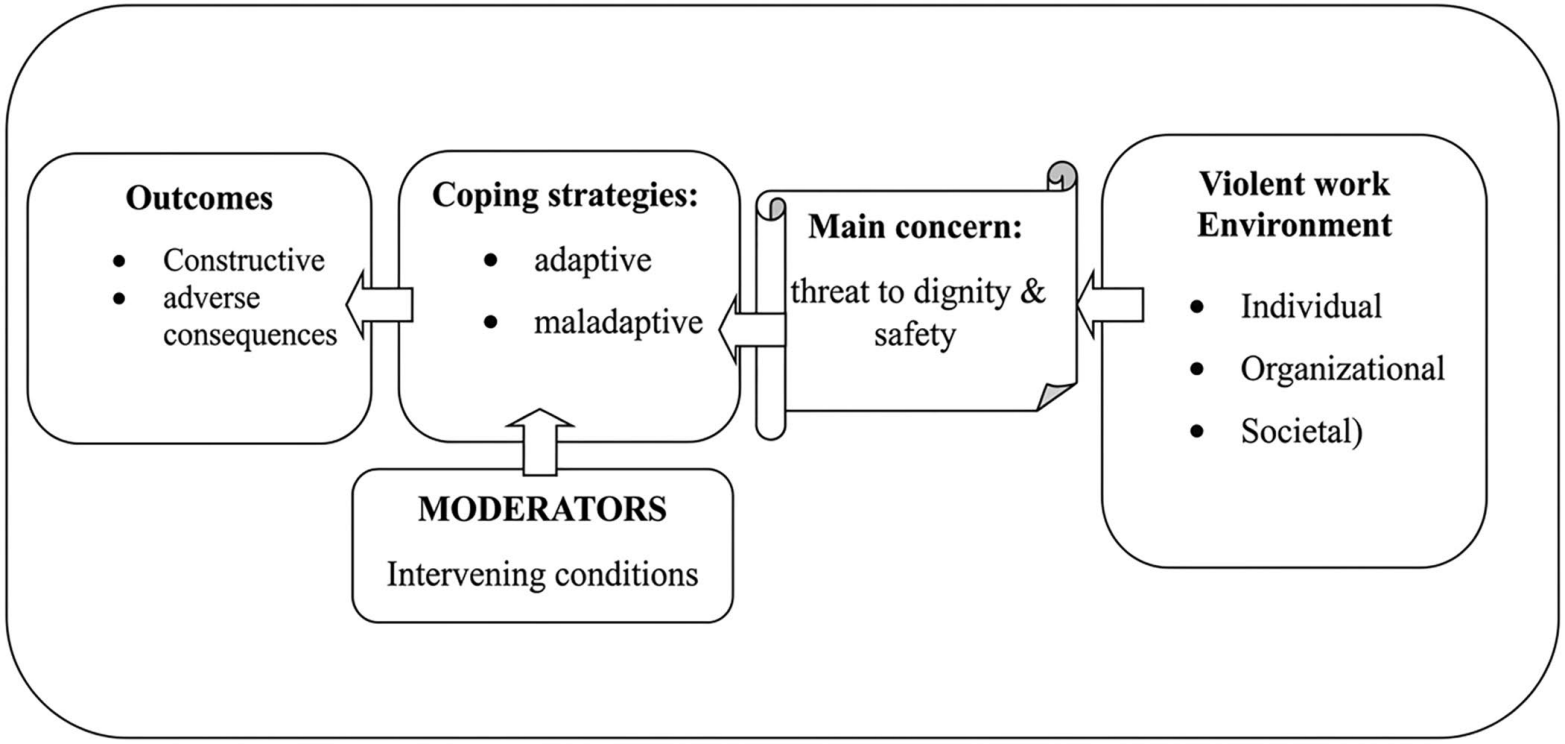
An illustration of the descriptive theory of protecting nurses' dignity and safety in the face of workplace violence.

The grounded theory conceptualises coping as a dynamic process centred on protecting dignity and safety, with contextual and intervening conditions shaping strategies that lead to either constructive or adverse outcomes (Figure 1).

### 5.2 Development of the Prescriptive Theory (Second Phase)

Building on the grounded theory, the second phase addressed how healthcare systems can strengthen adaptive coping while reducing harm. Using Walker and Avant's theory synthesis approach, the prescriptive theory ‘Promoting Nurses' Dignity and Safety at Work’ was developed by integrating inductive findings with existing literature to generate conceptually grounded guidance.

#### 5.2.1 Step 1: Conceptual Foundation

The core category—protecting nurses' dignity and safety—served as the conceptual anchor, integrating explanatory and guiding functions.

#### 5.2.2 Step 2: Integrative Literature Synthesis

Relevant literature was synthesised to identify interventions in four domains: safe environments, preventive education, supportive relationships and post‐incident recovery.

#### 5.2.3 Step 3: Model Construction and Prescriptive Logic

The prescriptive model translates the grounded theory into a conceptually oriented framework specifying suggested actions, by whom and at which level (individual, organisational and policy), emphasising principle‐based guidance rather than context‐specific procedures.

##### 5.2.3.1 Model Components

###### 5.2.3.1.1 Assumptions

*Assumptions*: Synthesised into four domains (person, nursing, health, environment), emphasising the dynamic relationship between dignity, coping and organisational responsibility.

###### 5.2.3.1.2 Relational Propositions

- Awareness of violence‐related risks enhances adaptive coping.
- Organisational support moderates negative impacts.
- Continuous evaluation sustains prevention efforts.

###### 5.2.3.1.3 Prescriptive Model Structure

The model integrates four paradigmatic concepts into a dynamic, iterative process guiding action to promote dignity and safety (Table 2).

**TABLE 2:** Paradigmatic and meta‐paradigmatic concepts.

Paradigmatic concepts	Core concept	Description
Core concept	Protecting Nurses' Dignity and Safety at Work	A continuous, adaptive organisational process that strengthens nurses' capacity to manage workplace violence through awareness, support and evaluation.
Context awareness	—	Systematic identification of workplace violence risks across individual, organisational and cultural levels to enhance preparedness.
Organisational support and empowerment	—	Provision of structural, educational and relational resources to strengthen adaptive coping and reduce harm.
Recovery and reinforcement	—	Post‐incident support mechanisms that promote emotional recovery, restore dignity and reinforce professional confidence.
Continuous evaluation	—	Ongoing monitoring and feedback processes to refine interventions and sustain effectiveness.

###### 5.2.3.1.4 Description of Meta‐Paradigmatic

Meta‐paradigmatic concepts emphasise nurses as active agents, nursing under conditions of risk and ethical strain, health as encompassing physical, psychological and social well‐being and environment as multi‐level and context‐sensitive, thereby prioritising conceptual transferability over procedural prescription.

###### 5.2.3.1.5 Goals and Objectives

The model aims to promote dignity and safety while reducing the consequences of workplace violence through:

- Strengthening preparedness
- Enhancing organisational accountability
- Supporting recovery
- Improving leadership engagement

### 5.3 Operationalisation of the Prescriptive Model

The model is operationalised through three interrelated processes:

1. Risk Recognition and Awareness.
2. Organisational Empowerment and Support.
3. Evaluation and Feedback.

Processes are iterative and context‐sensitive rather than linear, aligning with grounded theory findings and the model's conceptual nature. It should be noted that while these operational steps are directly informed by participant experiences and grounded theory findings, the model remains conceptually derived and requires further empirical testing to confirm effectiveness across diverse healthcare settings.

#### 5.3.1 Step 1: Contextual Risk Recognition and Awareness

Two integrated stages:

1. *Risk Identification*: Systematic assessment of environmental, patient‐related and systemic factors to inform conceptual strategies, not operational prescriptions.

A companion or patient who walks slowly, talks to himself or herself, or looks at you in a certain way is likely to be violent. (Participant 26).

2 *Awareness Building and Knowledge Translation*: Enhancing nurses' adaptive capacity through conceptual skill‐building (education, de‐escalation, stress management) and feedback loops.

The use of role‐playing techniques to manage violent situations was carried out by experienced supervisors in training classes was very useful. (Participant 15).

*Overall Aim*: Establish informed vigilance and proactive preparedness, transferable across healthcare contexts.

#### 5.3.2 Step 2: Empowerment Through Multilevel Organisational Support

Four integrated stages:

1. Maintaining a Safe and Healthy Work Environment: Conceptual focus on reducing triggers and fostering supportive structures.

Since they increased the number of male staff and especially put male staff on night shifts, the number of violent incidents has decreased a lot. (Participant 37).

2 Violence Prevention through Educational Interventions: Enhancing preparedness, skills and awareness.

They hold anger and stress management workshops for us. (Participant 11).

3 Providing Sympathetic Companionship and Respecting Dignity—relational and ethical support promoting resilience.

The psychological support and ethical behaviour of our head nurse were very encouraging to us. (Participant 23).

4 Nursing Rehabilitation Services Following Workplace Violence—post‐incident recovery and professional restoration.

A nurse who had been abused and traumatised was referred to a psychologist. (Participant 12).

*Overall Aim:* Translate awareness into empowerment through organisational, educational, relational and recovery‐oriented supports.

#### 5.3.3 Step 3 Evaluation and Feedback

Formative and summative evaluation with iterative refinement, ensuring conceptual guidance rather than prescriptive, fixed endpoints.

The periodic visits and feedback they received were very effective in improving the situation. (Participant 29).

### 5.4 Summary

**FIGURE 2:**
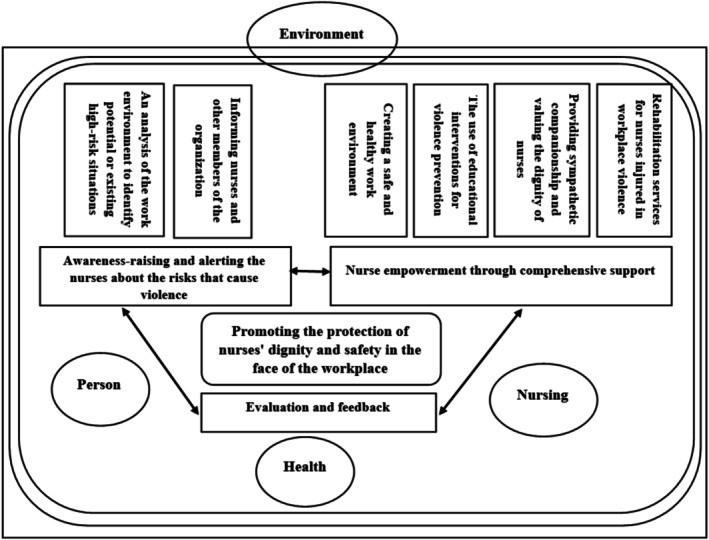
An illustration of the prescriptive model for promoting nurses ‘dignity and safety at work in the face of workplace violence (*Source:* Developed by the authors).

Figure 2 presents the final prescriptive model, structured around the three operational steps and framed by four nursing metaparadigms: person, nursing, health and environment. The model is conceptual, dynamic and context‐sensitive, highlighting continuous interaction between prevention, organisational support and evaluation to uphold nurses' dignity and safety. Bidirectional arrows indicate mutually reinforcing relationships among the operational steps, emphasising iterative adaptation rather than linear implementation.

## 6 Discussion

The primary purpose of this study was to explore the processes through which nurses cope with workplace violence and to develop a conceptually grounded prescriptive model that strengthens adaptive coping, reduces adverse psychological and professional outcomes and safeguards nurses' dignity and safety. The model, derived from our grounded theory findings, provides a structured framework for understanding how nurses experience, interpret and respond to workplace violence within multi‐level sociocultural and organisational contexts. By integrating individual, organisational and contextual dimensions of coping, this framework addresses a critical gap: existing models fail to comprehensively capture the dynamic interplay between personal coping, organisational response and culturally mediated factors.

This prescriptive model emphasises three key mechanisms:

1. *Strengthening adaptive coping strategies* through conceptual skill‐building, peer support and reflective learning.
2. *Modifying organisational culture and managerial responses* to support dignity and safety.
3. *Preserving professional dignity* through recognition, empowerment and relational support.

By grounding these mechanisms in nurses' real‐life experiences, the model achieves both conceptual depth and contextual validity, offering guidance that is adaptable across diverse healthcare settings.

Previous frameworks addressing workplace violence—such as Haddon's Matrix, the Context‐Mechanism‐Outcome (CMO) model, Critical Incident Stress Debriefing (CISD) and OSHA guidelines (Haddon Jr 1972; Antai‐Otong 2001; Occupational Safety and Health Administration 2015; Provost et al. 2021)—provide valuable insights but remain fragmented or limited in scope. None explicitly integrates nurses' cognitive, emotional and organisational coping processes while emphasising professional dignity in culturally sensitive contexts. Our model extends these prior approaches by offering a nursing‐specific, theory‐driven and empirically informed framework.

For instance, Spelten et al. (2020) applied Haddon's model to categorise preventive strategies across pre‐event, event and post‐event phases, focusing primarily on external interventions such as environmental modifications and reporting mechanisms (Spelten et al. 2020). In contrast, our model incorporates dynamic coping processes, highlighting the interaction between personal resilience, organisational support and dignity‐preserving mechanisms. This ensures that nurses are not only protected from violence but also supported in recovering emotionally and professionally.

Similarly, the CMO model (Provost et al. 2021) emphasises contextual influences on program outcomes. Our model builds on this by embedding continuous feedback and reflective learning loops, allowing healthcare institutions to adapt interventions based on nurses' lived experiences and evolving patterns of workplace violence, thus enhancing organisational adaptability and sustainability.

The CISD model (Antai‐Otong 2001) addresses psychological trauma through structured post‐incident debriefing but lacks preventive and systemic dimensions (Antai‐Otong 2001). Our model integrates both immediate support and long‐term preventive strategies, bridging individual recovery with organisational reform and promoting resilience before, during and after violent events.

Dignity‐promoting strategies (Combrinck et al. 2022) informed our model's focus on restoring and preserving professional dignity. The model integrates managerial, organisational and individual interventions that foster respect, empathy and moral recognition. It emphasises supportive communication channels and dignity‐based leadership practices, enabling nurses to rebuild confidence and maintain professional identity following incidents.

OSHA (2015) provides administrative and environmental control measures for violence prevention (Occupational Safety and Health Administration 2015). Our model complements these by addressing psychological and emotional dimensions, conceptualising coping as both a reactive and proactive competency that mediates well‐being, performance and adaptive capacity.

The strength of our prescriptive model lies in its grounding in qualitative evidence from Iranian nurses, respecting cultural values such as collective responsibility, moral integrity and interpersonal harmony, while aligning with global standards for occupational safety and dignity in nursing. The rigour of the model is supported by grounded theory procedures (Corbin and Strauss 2015), including theoretical sampling, constant comparative analysis and memoing, ensuring that conceptual categories emerged inductively. Diverse participants (nurses, head nurses, supervisors, educators) enhanced credibility, dependability and transferability through theoretical saturation.

From a theoretical perspective, the model bridges coping theory (Lazarus and Folkman 1985) with nursing systems theory (Meleis 2018), linking individual adaptation with organisational transformation (Lazarus and Folkman 1985; Meleis 2018). Practically, it provides healthcare leaders and policymakers with principled guidance for interventions, policies and education to protect nurses' dignity and safety while reducing workplace violence.

Future research should focus on empirical testing and validation across diverse cultural and organisational settings, including longitudinal and implementation studies, to refine and confirm the model's effectiveness and generalisability.

### 6.1 Strengths and Limitations of the Work

*Strengths:*

- Contextually grounded yet theoretically transferable prescriptive model.
- Systematic integration of inductive findings with established theory.
- Multi‐stakeholder perspectives ensuring rich data and saturation.

*Limitations:*

- Findings are shaped by the cultural, organisational and temporal context.
- Participants may have moderated responses due to professional concerns.
- Operationalisation may require adaptation in different settings with varying resources, regulations, or cultural norms.
- The model remains conceptually derived and requires empirical validation before claims of effectiveness can be generalised.

### 6.2 Recommendations for Further Research

- Empirical testing and refinement of the model across diverse clinical and cultural settings.
- Mixed‐methods and quasi‐experimental studies to evaluate effectiveness in reducing workplace violence and improving coping and well‐being.
- Development and validation of standardised measurement tools based on model components.
- Longitudinal studies to assess sustainability and explore the influence of organisational culture and leadership styles.

### 6.3 Implications for Policy and Practice

*Clinical practice:*

- Integrate the model into routine risk assessment, violence prevention and post‐incident response.
- Implement reporting systems, multidisciplinary committees and continuous evaluation.

*Management and Policy‐Making:*

- Institutionalise the model through enforceable policies, mandatory training and accountability frameworks.
- Systematically detect, document and monitor violent incidents, reviewing safety and staffing practices.
- Recognise nurses' dignity and safety as ethical and professional imperatives.

*Nursing Education:*

- Embed the model in undergraduate and postgraduate curricula.
- Include content on workplace violence, ethical practice, legal rights, emotional regulation and adaptive coping.
- Prepare future nurses for high‐risk clinical environments with both theoretical and practical training.

## 7 Conclusion

This study advances nursing knowledge by developing a conceptually robust, prescriptive, theory‐informed model addressing how nurses can maintain dignity and safety amid persistent workplace violence. Grounded in nurses' lived experiences and extended through systematic theory synthesis, the Model of Promoting Nursing Dignity and Safety moves beyond descriptive accounts, explicating how adaptive coping can be fostered and sustained through multilevel organisational interventions.

Theoretically, the model identifies dignity and job security as central processes, extending workplace violence and occupational stress frameworks. Unlike models focused solely on individual resilience, it highlights the dynamic interplay between individual, organisational and environmental factors, aligning nursing theory with workforce shortages, moral distress and unsafe clinical environments.

Practically, the model provides a transferable, context‐sensitive framework integrating prevention, empowerment, relational support and evaluation into a continuous cycle. It equips leaders and educators with principled guidance to strengthen professional identity, enhance workplace safety and support sustainability of the nursing workforce globally.

## Author Contributions

The research team monitored the progress of the study at each stage. The authors reviewed and approved all data collection and analysis procedures. Z.E.R., P.M.S., F.A. and A.R. contributed to the final written report's composition, review and correction.

## Funding

The authors have nothing to report.

## Conflicts of Interest

The authors declare no conflicts of interest.

## Data Availability

The data that support the findings of this study are available from the corresponding author upon reasonable request.
